# Ordinal state‐trait regression for intensive longitudinal data

**DOI:** 10.1111/bmsp.12285

**Published:** 2022-09-08

**Authors:** Prince P. Osei, Philip T. Reiss

**Affiliations:** ^1^ School of Mathematics and Statistics Carleton University Ottawa Ontario Canada; ^2^ Department of Statistics University of Haifa Haifa Israel

**Keywords:** cumulative logistic regression, experience sampling, latent response, ordinal mixed‐effects model, quadratic penalty

## Abstract

In many psychological studies, in particular those conducted by experience sampling, mental states are measured repeatedly for each participant. Such a design allows for regression models that separate between‐ from within‐person, or trait‐like from state‐like, components of association between two variables. But these models are typically designed for continuous variables, whereas mental state variables are most often measured on an ordinal scale. In this paper we develop a model for disaggregating between‐ from within‐person effects of one ordinal variable on another. As in standard ordinal regression, our model posits a continuous latent response whose value determines the observed response. We allow the latent response to depend nonlinearly on the trait and state variables, but impose a novel penalty that shrinks the fit towards a linear model on the latent scale. A simulation study shows that this penalization approach is effective at finding a middle ground between an overly restrictive linear model and an overfitted nonlinear model. The proposed method is illustrated with an application to data from the experience sampling study of Baumeister et al. (2020, *Personality and Social Psychology Bulletin*, 46, 1631).

## INTRODUCTION

In experience sampling and other intensive longitudinal studies, mental state variables are repeatedly collected for each participant. Multilevel regression models for data of this type allow one to separate between‐ from within‐person components of association between a predictor variable *x* and a response variable *y*, both representing mental states. These models, however, are generally designed for continuous variables. Since mental states are most often measured on an ordinal scale, there is a need to develop analogous models for ordinal responses and predictors. Meeting that need is the primary aim of this paper.

Suppose, for example, that *x* is a measure of mental focus, while *y* denotes reported happiness. A repeated‐measures design allows us to study the relationship between *x* and *y* on both the between‐person and the within‐person level. In our example, the former means we ask whether people who are more focused on average will tend to be happier on average; the latter entails asking whether a given person, in moments of greater focus, will tend to be happier.

Suppose the sample consists of *I* individuals, where the *i*th person is observed *J*_*i*_ times. A basic linear mixed‐effects model for disentangling between‐ and within‐person effects (e.g., Neuhaus & Kalbfleisch, 1998; Wang & Maxwell, 2015) may be written as

(1) $$y_{\mathrm{ij}}=\beta_{0}+\beta_{1}{\overset{―}{x}}_{i\cdot }+\beta_{2}\left(x_{\mathrm{ij}}-{\overset{―}{x}}_{i\cdot }\right)+u_{i}+\varepsilon_{\mathrm{ij}},$$

for $i\in \left\{1,\ldots ,I\right\},j\in \left\{1,\ldots ,J_{i}\right\}$, where $y_{\mathrm{ij}},x_{\mathrm{ij}}$ are the values of *y,x* for the *j*th observation of the *i*th individual; ${\bar{x}}_{i\cdot }$ is the mean of all *x* values for the *i*th individual; $u_{i}$ is the random effect of the *i*th individual (for simplicity, we consider here only random intercepts but not random slopes); and $\varepsilon_{\mathrm{ij}}$ denotes error. In the standard specification, the random effects are usually assumed independent and identically distributed (i.i.d.) as $N\left(0,\sigma_{u}^{2}\right)$ for some $\sigma_{u}^{2}>0$, and the errors are i.i.d. $N\left(0,\sigma_{\varepsilon }^{2}\right)$ for some $\sigma_{\varepsilon }^{2}>0$, with mutual independence of the random effects and errors.

As noted by Curran and Bauer (2011), statistical models of this type have a long history of application in sociology and education, for disaggregating between‐ from within‐*group* effects (e.g., Cronbach & Webb, 1975; Firebaugh, 1978; Raudenbush & Willms, 1995). In the psychological context, following Zilcha‐Mano and Errázuriz (2015), we may think of ${\overset{―}{x}}_{i\cdot }$ as a ‘trait‐like’ measure of the variable *x*, whereas *x*_*ij*_ is ‘state‐like’, and thus $x_{\mathrm{ij}}-{\bar{x}}_{i\cdot }$ represents the deviation of the momentary state from individual *i*’s stable trait. More formal quantitative definitions of states and traits can be found, for example, in Steyer et al. (2015).

In model (1), $\beta_{1},\beta_{2}$ can be interpreted as between‐person (trait) and within‐person (state) effects, respectively. In other words, *β*_1_ can be viewed as the effect of ${\bar{x}}_{i\cdot }$ on the person‐*i* mean response ${\overset{―}{y}}_{i\cdot }$, as can be seen by averaging (1) over *j* to obtain

$${\bar{y}}_{i\cdot }=\beta_{0}+\beta_{1}{\bar{x}}_{i\cdot }+u_{i}+{\bar{\varepsilon }}_{i},$$

where ${\bar{\varepsilon }}_{i\cdot }$ is the person‐*i* mean error. Subtracting this equation from (1) yields

$$y_{\mathrm{ij}}-{\bar{y}}_{i\cdot }=\beta_{2}\left(x_{\mathrm{ij}}-{\bar{x}}_{i\cdot }\right)+\left(\varepsilon_{\mathrm{ij}}-{\bar{\varepsilon }}_{i\cdot }\right),$$

and this supports viewing *β*_2_ as a within‐person effect: specifically, the effect of the deviation of *x* from its within‐person mean, on the deviation of *y* from *its* within‐person mean.

## MOTIVATING DATA SET

In this section we introduce the data set that motivated our work, and briefly discuss why the ordinal nature of the variables of interest renders model (1) ill‐suited to these data.

Baumeister et al. (2020) report on two experience sampling studies to investigate ‘mental time travel’ – thoughts that are oriented towards the past and future as opposed to the present. Here, rather than focusing on time orientation, we examine a series of questions posed at each contact in the authors' Study 1. The data set, which is publicly available at https://osf.io/9uytp/, includes 491 participants who were contacted up to six times per day for each of three consecutive days, for a total of 6685 observations (contacts). Near the end of the survey, participants were asked five questions about their current thoughts and states, each rated on a 7‐point scale. The following are the questions, each one preceded by the corresponding variable name to be used below:

1. *Pleasant*. Altogether, to what extent were your thoughts about something pleasant/unpleasant? (from very unpleasant (–3) to very pleasant (+3))
2. *Absorbed*. How involved are you with what is happening right now? (from totally detached [0] to totally absorbed [6])
3. *Satisfied*. How satisfied with your life are you right now? (from not at all [0] to very much [6])
4. *Exhausted*. How mentally exhausted do you feel right now? (from not at all [0] to very much [6])
5. *Stressed*. How stressed are you right now? (from not at all [0] to very much [6])

We are interested in examining associations among these five variables, while disentangling the state‐like and trait‐like (within‐ and between‐person) components of such associations. Consider, for example, Table 1, in which we cross‐tabulate the variables pleasant and stressed, with all non‐missing observations for all 491 participants pooled together. There is a clear negative relationship between the two variables: a standard chi‐squared test would very resoundingly reject the hypothesis that they are independent, although we do not report such a test result since the chi‐squared test does not take into account the nesting of observations within participants. Does this negative relationship mean that chronically stressed people have a general tendency to think unpleasant thoughts (what we might call a trait‐driven association)? Alternatively, does it means that in moments of being stressed people's thoughts tend to be less pleasant (a state‐driven association)? Or if both of these patterns hold, how might we quantify their relative contributions to the overall negative relationship?

**TABLE 1:** Cross‐tabulation of pleasantness of thoughts against stress, with all observations for all 491 participants pooled together

Pleasant	Stressed						
	0	1	2	3	4	5	6
–3	16	14	49	160	323	353	408
–2	7	23	71	143	346	358	132
–1	7	42	144	206	475	336	91
0	4	35	98	261	233	128	37
1	13	72	210	222	318	143	41
2	30	77	152	79	134	86	16
3	70	82	93	46	64	29	39

We might try using model (1) to answer these questions. Note, however, that this model is intended for continuous *y* and *x*, whereas in our setting these variables are ordinal. There are at least two major problems with applying this model with ordinal *y*:

1. A continuous‐data model such as (1) treats the differences between adjacent categories as equal, which may not be appropriate.
2. Standard likelihood‐based fitting of the mixed‐effects model assumes that the random effects *u*_*i*_ and errors $\varepsilon_{\mathrm{ij}}$ are normally distributed. For ordinal *y*, the normality assumption cannot hold, at least not for the errors.

Liddell and Kruschke (2018) offer a detailed discussion of the pitfalls of applying continuous‐data models to ordinal data, with emphasis on examples in social psychology. Section Ordinal predictor offers a further critique of model (1) given that *x* is also ordinal.

## EXTENSION TO ORDINAL DATA

We now present an alternative to model (1) that is appropriate for settings such as our motivating application, in which (i) the response *y* is ordinal and (ii) the predictor *x* is also ordinal.

### Ordinal response

Regression for an ordinal response $y\in \left\{1,2,\ldots ,M_{y}\right\}$ (e.g., Agresti, 2010) is often formulated in terms of an underlying continuous latent response ℓ, by positing a sequence of $M_{y}-1$ cutpoints $\theta_{1}\leq \ldots \leq \theta_{M_{y}-1}$ such that the manifest response *y* is determined by the value of ℓ relative to the cutpoints:

(2) $$y=\left\{\begin{array}{ll} 1 & \text{if}\;\ell <\theta_{1},\\ 2 & \text{if}\;\theta_{1}<\ell <\theta_{2},\\ \vdots & \\ M_{y} & \text{if}\;\ell >\theta_{M_{y}-1}. \end{array}\right.$$

If we take $\theta_{0}=-\infty$ and $\theta_{M_{y}}=\infty$, the above can be written simply as y=m if $\theta_{m-1}<\ell <\theta_{m}$, for each $m\in \left\{1,\ldots ,M_{y}\right\}$. This provides a natural way to extend model (1) from continuous to ordinal response: we simply replace *y* on the left‐hand side with the continuous latent ℓ, yielding

(3) $$\ell_{\mathrm{ij}}=\beta_{0}+\beta_{1}{\bar{x}}_{i\cdot }+\beta_{2}\left(x_{\mathrm{ij}}-{\bar{x}}_{i\cdot }\right)+u_{i}+\varepsilon_{\mathrm{ij}}.$$

Here the $u_{i}$ are i.i.d. $N\left(0,\sigma_{u}^{2}\right)$ and independent of the i.i.d. $\varepsilon_{\mathrm{ij}}$ as before, but there are two standard choices for the latent error distribution, each of which gives rise to a different (conditional) distribution for the random ordinal response *Y*_*ij*_:

1. If the $\varepsilon_{\mathrm{ij}}$ are drawn from the standard normal distribution, then the latent model (3), linked to the ordinal response by (2), is equivalent to the cumulative probit model

$$\Phi^{-1}\left[\;\mathrm{Pr}\;\left(Y_{\mathrm{ij}}\leq m\right)\right]=\theta_{m}-\beta_{0}-\beta_{1}{\bar{x}}_{i\cdot }-\beta_{2}\left(x_{\mathrm{ij}}-{\bar{x}}_{i\cdot }\right)-u_{i},$$

where Φ is the standard normal cumulative distribution function.

2 If the $\varepsilon_{\mathrm{ij}}$ are drawn from a standard logistic distribution (i.e., with mean 0 and scale parameter 1, implying variance $\pi^{2}/3$), then latent model (3) is equivalent to the cumulative logistic model

(4) $$\text{logit}\;\mathrm{Pr}\;\left(Y_{\mathrm{ij}}\leq m\right)\equiv \log\frac{\;\mathrm{Pr}\;\left(Y_{\mathrm{ij}}\leq m\right)}{\;\mathrm{Pr}\;\left(Y_{\mathrm{ij}}>m\right)}=\theta_{m}-\beta_{0}-\beta_{1}{\bar{x}}_{i\cdot }-\beta_{2}\left(x_{\mathrm{ij}}-{\bar{x}}_{i\cdot }\right)-u_{i},$$

which in turn is equivalent to

$$\;\mathrm{Pr}\;\left(Y_{\mathrm{ij}}\leq m\right)=\frac{1}{1+\exp\left[\beta_{0}+\beta_{1}{\bar{x}}_{i\cdot }+\beta_{2}\left(x_{\mathrm{ij}}-{\bar{x}}_{i\cdot }\right)+u_{i}-\theta_{m}\right]}.$$

We pursue the latter approach, cumulative logistic regression, because the resulting coefficients can be interpreted as log‐odds ratios, and also for computational convenience: in our particular setting the cumulative logistic model makes available the implementation described below in Section Optimal selection of the shrinkage parameter.

As we discuss next, when *x* is also ordinal (as in our motivating data set), a modification to model (3)/(4) is warranted.

### Ordinal predictor

The model of the previous subsection takes into account the ordinal nature of the response *y*. In our application of interest the predictor *x* is also ordinal, taking values $1,\ldots ,M_{x}$, say. But the above model treats *x* as continuous, in the sense that a unit change in ${\bar{x}}_{i\cdot }$ or in $x_{\mathrm{ij}}-{\bar{x}}_{i\cdot }$ has a constant effect (of $\beta_{1}$ or $\beta_{2}$, respectively) on the expected latent response. Indeed, using the mean ${\bar{x}}_{i\cdot }$ as a person‐specific average is in itself more appropriate for a continuous than for an ordinal *x*. If both *y* and *x* are ordinal, it is inconsistent to treat *y* as ordinal but proceed as if *x* were continuous. This motivates a novel model that we describe next.

To begin with, instead of defining the person‐specific average as the mean ${\bar{x}}_{i\cdot }$, which is typically not a possible value for ordinal *x*, we use the median ${\tilde{x}}_{i}$. Our proposed model for the latent response, instead of (3), is then

(5) $$\ell_{\mathrm{ij}}=\alpha +\tau_{{\tilde{x}}_{i}}+\gamma_{{\tilde{x}}_{i},x_{\mathrm{ij}}}+u_{i}+\varepsilon_{\mathrm{ij}},$$

where $\tau_{r}$ is the effect of having median *x* equal to *r*, while $\gamma_{r,s}$ is the effect of having median *x* equal to *r* and current value equal to *s*. For example, for a person with median value 3 and current value 4 of *x*, the latent response has expected value $\alpha +\tau_{3}+\gamma_{3,4}$ for that observation. For identifiability, we set

(6) $$\tau_{\tilde{x}}=0\;\text{and}\;\gamma_{r,r}=0\;\text{for}\;\text{each}\;r,$$

where $\tilde{x}$ is the median of *x* for the entire data set. Thus the intercept *α* is the expectation of the latent response for an observation with $x=\overset{―}{x}$, for a person whose median value is also $\tilde{x}$.

The model specification is completed exactly as in Section Ordinal response, with the latent‐scale random effects $u_{i}$ having a normal distribution, the latent errors $\varepsilon_{\mathrm{ij}}$ having a standard logistic distribution, and the value of $y_{\mathrm{ij}}$ determined by the value of $\ell_{\mathrm{ij}}$ relative to cutpoints $\theta_{1}\leq \ldots \leq \theta_{M_{y}-1}$. Then, analogously to (4), the latent model (5) gives rise to the cumulative logistic model

(7) $$\text{logit}\;\mathrm{Pr}\;\left(Y_{\mathrm{ij}}\leq m\right)=\theta_{m}-\alpha -\tau_{{\tilde{x}}_{i}}-\gamma_{{\tilde{x}}_{i},x_{\mathrm{ij}}}-u_{i}.$$

To render this model identifiable, a constraint must be imposed on the θs. Our particular implementation (see Section Optimal selection of the shrinkage parameter) adopts the constraint $\theta_{1}=-1$.

### An alternative formulation of model (5)

Model (5) can equivalently be written as

(8) $$\ell_{\mathrm{ij}}=f\left({\tilde{x}}_{i},x_{\mathrm{ij}}\right)+u_{i}+\varepsilon_{\mathrm{ij}},$$

where

(9) $$f\left(r,s\right)\equiv E\left(\ell_{\mathrm{ij}}|{\tilde{X}}_{i}=r,X_{\mathrm{ij}}=s\right),$$

is an arbitrary function on K, the set of all trait‐state pairs occurring in the data – that is,

(10) $$K=\left\{\left(r,s\right)\in {\left\{1,\ldots ,M_{x}\right\}}^{2}:\left({\tilde{x}}_{i},,x_{\mathrm{ij}}\right)=\left(r,s\right)\;\text{for}\;\text{some}\;i,j\right\}.$$

Formula (8) has several advantages over (5): it makes more explicit the model's generality, and is simpler to work with for defining a penalty (see Section A penalization strategy) and for specifying a true model (as in the simulation study in Section Simulation study). An advantage of (5) is that it explicitly decomposes $f\left(r,s\right)$ into interpretable parameters: $\alpha =f\left(\tilde{x},\tilde{x}\right)$, an increment $\tau_{r}$ attributed to the trait level (person median) of *x*, and an increment $\gamma_{r,s}$ attributed to the current state of *x*.

If the model is given in form (8) for some function $f\left(\cdot ,\cdot \right)$, it is straightforward to derive the equivalent model with parameters $\alpha ,\tau_{r},\gamma_{r,s}$ as in (5). By the identifiability conditions (6),

$$\begin{array}{l} f\left(\tilde{x},\tilde{x}\right)=\alpha ,\\ f\left(r,r\right)=\alpha +\tau_{r}\;\text{for}\;\mathrm{all}\;r,\\ f\left(r,s\right)=\alpha +\tau_{r}+\gamma_{r,s}\;\text{for}\;\mathrm{all}\;r,s; \end{array}$$

thus the model (5) parameters $\tau_{r},\gamma_{r,s}$ for all *r*,*s* are given by

(11) $$\tau_{r}=f\left(r,r\right)-f\left(\tilde{x},\tilde{x}\right),\;\gamma_{r,s}=f\left(r,s\right)-f\left(r,r\right).$$

### Connection with the standard model

To see how model (5) is a natural extension of (3) (which in turn is the latent‐response analogue of the standard model (1)), suppose there exist $\beta_{1},\beta_{2}$ such that

(i) $\tau_{r}=\beta_{1}\left(r-\tilde{x}\right)$ for each *r*, that is, $\tau_{r}$ is proportional to the deviation of person median *r* from global median $\tilde{x}$; and

(ii) $\gamma_{r,s}=\beta_{2}\left(s-r\right)$ for each r,s, that is, $\gamma_{r,s}$ is proportional to the deviation of level *s* from person median *r*.

Then (5) reduces to

(12) $$\ell_{\mathrm{ij}}=\beta_{0}+\beta_{1}{\tilde{x}}_{i}+\beta_{2}\left(x_{\mathrm{ij}}-{\tilde{x}}_{i}\right)+u_{i}+\varepsilon_{\mathrm{ij}},$$

where $\beta_{0}=\alpha -\beta_{1}\tilde{x}$; this is just (3) but with the person median ${\tilde{x}}_{i}$ replacing the mean ${\bar{x}}_{i}$. Model (5) extends (12) by relaxing the proportionality assumptions (i), (ii) above.

In terms of the formulation of Section An alternative formulation of model (5), equation (12) is just the special case of (8) with $f\left(r,s\right)=\beta_{0}+\beta_{1}r+\beta_{2}\left(s-r\right)$. Equivalently, with the simple reparametrization $\beta_{0}^{*}=\beta_{0}$, $\beta_{1}^{*}=\beta_{1}-\beta_{2}$, $\beta_{2}^{*}=\beta_{2}$, we may say that if *f* has the linear form

(13) $$f\left(r,s\right)=\beta_{0}^{*}+\beta_{1}^{*}r+\beta_{2}^{*}s,$$

then the general model (8) reduces to (12). In the next section we argue that allowing an arbitrary $f\left(r,s\right)$ may render the model overparametrized whereas requiring *f* to be linear may make it underparametrized, and we propose a compromise solution.

## A PENALIZATION STRATEGY

### Motivation

Model (12), a latent‐scale analogue of the standard model (1) for disaggregating between‐ and within‐person effects, has three fixed‐effect coefficients. At the opposite extreme, our proposed latent‐scale model (5) has ∣K∣ fixed‐effect coefficients, where K is defined in (10) and ∣K∣ denotes its cardinality. In other words, model (5) has as many fixed effects as the number of trait‐state combinations occurring in the data, which may be as high as $M_{x}^{2}$, the square of the number of levels of *x*. This more general model allows for nonlinear, and possibly non‐monotonic, effects of *x* on the latent variable ℓ at either the state or the trait level. Given the number of observations in typical experience sampling studies, ∣K∣ may be a reasonable number of parameters to estimate. On the other hand, especially for rare trait‐state combinations $\left(r,s\right)$, estimation of $\gamma_{r,s}$ is expected to be unstable (although this can be mitigated in practice by merging of pairs, which reduces the number of coefficients; see the Appendix). In other words, the proposed model may be overparametrized.

A classical approach to stabilizing estimation in overparametrized models is ridge regression (Hoerl & Kennard, 1970). In its generalized version, given a model with coefficient vector ζ and log‐likelihood *l*(ζ), ridge regression maximizes

(14) $$\;l\left(\zeta \right)-\frac{\lambda }{\;2}\;\zeta^{T}P\zeta ,$$

where **P** is a ‘penalty’ matrix such that, for the specific problem at hand, it is appropriate to shrink the estimate of ζ towards values for which the penalty functional $\;\zeta^{T}P\zeta$ is zero; and λ is a tuning parameter that determines the extent of this shrinkage (optimal data‐driven choice of λ is discussed in Section Optimal selection of the shrinkage parameter). In our case ζ consists of α, the $\tau_{r}$, and the $\gamma_{r,s}$ with r≠s (recall that by (6), $\gamma_{r,r}=0$ for each *r*). In light of the above discussion, it is natural to choose P such that the estimates are shrunk towards the more restrictive and more traditional model (12), or equivalently *f* is shrunk towards a linear function (13). Thus for small λ the result will be similar to fitting the ∣K∣‐fixed‐effects model (5)/(8) in an unconstrained manner; for large λ it will be close to the three‐fixed‐effects model (12). The next subsection explains how to define such a matrix **P**.

### Defining the penalty

To choose a penalty matrix **P** such that maximizing (14) shrinks the estimate of $f\left(r,s\right)$ towards a linear function, we borrow an idea from spline smoothing. There one typically estimates a function on a continuous domain by imposing a penalty, as in (14), that shrinks the fit towards a space of polynomial functions. Specifically, the method of thin‐plate splines (Green & Silverman, 1994; Wahba, 1990) estimates a smooth bivariate function $f\left(r,s\right)$ by maximizing a criterion of the form (14) where ζ and **P** are chosen so that

(15) $$\;\zeta^{T}P\zeta =J\left(f\right)=\int \int \left[{\left(\frac{\partial^{2}f}{\partial r^{2}}\right)}^{2}+2{\left(\frac{\partial^{2}f}{\partial r\partial s}\right)}^{2}+{\left(\frac{\partial^{2}f}{\partial s^{2}}\right)}^{2}\right]\text{drds}.$$

Since $J\left(f\right)=0$ if and only if *f* is a linear function of *r*,*s*, the effect of this penalty is to shrink the estimate of *f* towards the set of such functions.

For our setting, in which $r,s\in \left\{1,\ldots ,M_{x}\right\}$, it is natural to shrink towards linear functions by means of a discrete analogue of (15):

(16) $$J^{*}\left(f\right)=\sum {\left[\Delta_{\mathrm{rr}}f\left(r,s\right)\right]}^{2}+2\sum {\left[\Delta_{\mathrm{rs}}f\left(r,s\right)\right]}^{2}+\sum {\left[\Delta_{\mathrm{ss}}f\left(r,s\right)\right]}^{2},$$

where

(17) $$\Delta_{\mathrm{rr}}f\left(r,s\right)=f\left(r+1,s\right)-2f\left(r,s\right)+f\left(r-1,s\right),$$

(18) $$\Delta_{\mathrm{rs}}f\left(r,s\right)=f\left(r+1,s+1\right)-f\left(r+1,s\right)-f\left(r,s+1\right)+f\left(r,s\right),$$

(19) $$\Delta_{\mathrm{ss}}f\left(r,s\right)=f\left(r,s+1\right)-2f\left(r,s\right)+f\left(r,s-1\right).$$

$\Delta_{\mathrm{rr}}$ can be thought of as the second‐order difference in *f* with respect to *r* for given *s*; $\Delta_{\mathrm{ss}}$ is the second‐order difference with respect to *s* for given *r*; and $\Delta_{\mathrm{rs}}$ is the ‘mixed’ second‐order difference. In (16), each sum is taken over all r,s for which the summands are defined. This means that the two arguments of *f* in each summand in (17), (18) or (19) refer to a trait‐state pair in ${\left\{1,\ldots ,M_{x}\right\}}^{2}$ for which there are observations in the data, so that *f* is defined for that pair of arguments; recall that *f* is defined only on the set K given in (10). Details of how to define **P** so that $\;\zeta^{T}P\zeta =J^{*}\left(f\right)$ are given in the Appendix.

It is easy to show that $J^{*}\left(f\right)=0$ when *f* is linear as in (13). When $K={\left\{1,\ldots ,M_{x}\right\}}^{2}$ the converse holds: $J^{*}\left(f\right)=0$ implies *f* is linear. In our experience, even when K is a proper subset of ${\left\{1,\ldots ,M_{x}\right\}}^{2}$, $J^{*}\left(f\right)=0$ holds only for linear *f*. Thus the penalty functional (16) shrinks towards linear functions *f*, or equivalently towards the conventional model (12).

Extensive discussion regarding penalties for ordinal predictors can be found in Gertheiss and Tutz (2009) and Tutz and Gertheiss (2016). The penalty functional (16), which is specifically designed for our state‐trait regression model, is novel.

### Optimal selection of the shrinkage parameter

In the smoothing literature, from which our penalty was derived, the aim of automatic selection of the tuning parameter λ in (14) is usually either to minimize a prediction error criterion or to maximize a likelihood. The latter approach, advocated by Ruppert et al. (2003), Reiss and Ogden (2009) and Wood (2011) among others, posits a model in which the shrunken parameters are random effects; λ is then a parameter related to the variance of these effects, which is one of the parameters over which the likelihood is maximized. Since our model already incorporates random effects, namely $u_{i}$, it is particularly convenient and natural to adopt a random‐effects/likelihood approach to selecting λ.

Such an approach to selection of λ is made possible for our model by the penalized regression package *mgcv* (Wood, 2017) for R (R Core Team, 2021), based on two seminal contributions. First, Wood (2011) derived a Laplace approximation to (14) for semiparametric generalized linear models, in which the log‐likelihood may be replaced by a restricted log‐likelihood (Laird & Ware, 1982), and showed how to maximize the approximate criterion with respect to both λ and ζ. Subsequently, Wood et al. (2016) extended the methods of *mgcv* to a broader class of models, including cumulative logistic (mixed) models.

Another R package that can fit ordinal mixed models is *ordinal* (Christensen, 2019), which is more flexible than *mgcv* in terms of the available link functions (e.g., probit rather than logit) and random‐effects structure. Our default implementation, however, employs *mgcv* since it can incorporate the above custom‐designed penalty.

## PLEASANT THOUGHTS AND STRESS REVISITED

**FIGURE 1:**
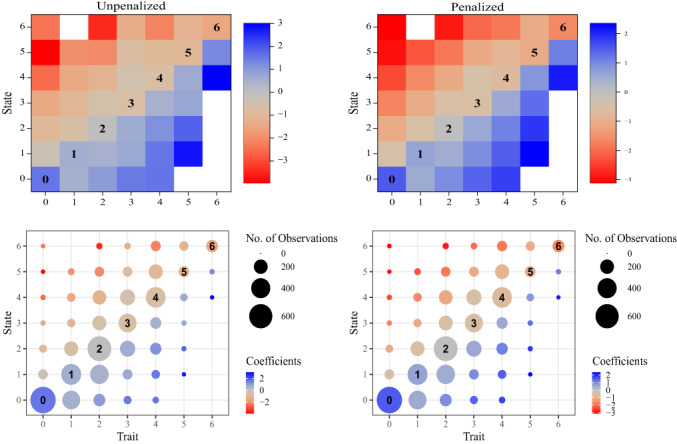
Parameter estimates for the latent model (5) regressing pleasantness (of thoughts) on stress. Upper row: Shown along the main diagonal (squares labelled 0,1,…,6) are the parameter estimates ${\hat{\tau }}_{0},\ldots ,{\hat{\tau }}_{6}$. Off‐diagonal squares are parameter estimates ${\hat{\gamma }}_{r,s}$ for r≠s. At left, estimates from unpenalized model; at right, from penalized model described in Section A penalization strategy. Lower row: The same parameter estimates presented in bubble plots, with bubble size indicating the number of observations for each trait‐state combination.

We now apply model (5) to re‐examine the negative relationship, suggested by Table 1, between pleasantness (of thoughts) and stress for the data of Baumeister et al. (2020). Regressing pleasant on stress, we obtain the parameter estimates for model (5) that are displayed in the upper left‐hand panel of Figure 1. Shown along the main diagonal (squares labelled 0, 1, …,6) are the parameter estimates ${\hat{\tau }}_{0},\ldots ,{\hat{\tau }}_{6}$ representing ‘trait effects’ of participants' typical stress levels, as estimated by their person‐specific medians. Off‐diagonal squares are parameter estimates ${\hat{\gamma }}_{r,s}$ for r≠s, representing the ‘state effects’ of being more or less stressed than usual. In this case the overall median of stress is 2, and hence, as discussed in Section Ordinal predictor, we fix $\tau_{2}=0$. Thus the positive values of ${\hat{\tau }}_{0},{\hat{\tau }}_{1}$ and the negative values of ${\hat{\tau }}_{3},{\hat{\tau }}_{4},{\hat{\tau }}_{5},{\hat{\tau }}_{6}$ suggest that people with a general tendency to be stressed also tend to think less pleasant thoughts – a negative trait‐level association. Inspection of the off‐diagonal squares reveals that in general, ${\hat{\gamma }}_{r,s}<0$ when r<s, while ${\hat{\gamma }}_{r,s}>0$ when r>s. This can be interpreted as a negative state‐level association: being more exhausted than usual (trait *r* and state *s* with *r* < *s*) is associated with less pleasant thoughts. For brevity we do not list all these coefficients along with confidence intervals and *p*‐values, but we note that *p* < .0001 (uncorrected) for most of them.

We observe, then, a negative relationship between pleasant thoughts and stress at both the trait and state levels. But in some cases, a plot of this type could reveal an instance of Simpson's paradox (see Kievit et al., 2013), with a positive state association but a negative trait association or vice versa.

In the lower left‐hand panel of Figure 1, the same estimates ${\hat{\tau }}_{r}$ and ${\hat{\gamma }}_{r,s}$ are presented in the form of bubble plots, where the bubble size corresponds to the number of observations with the given state‐trait combination. The bubble plot has the advantage of emphasizing that some estimates are for uncommon state‐trait combinations, and hence are relatively uncertain and should be treated with caution.

The right‐hand panels of Figure 1 display estimates from the corresponding penalized model. Note that if *f* were linear as in (13), it would follow from (11) that $\gamma_{r,s}\propto s-r$, so that the values on each off‐diagonal band (from lower left to upper right) would be constant. Thus our penalty, which shrinks towards linear *f*, results in more uniform values within each off‐diagonal band, as can be seen by comparing the left‐ and right‐hand panels of Figure 1.

## SIMULATION STUDY

### Study design

We conducted a simulation study to compare the performance of the proposed penalized fitting of model (8) with the two competitors described above: (i) the restricted model (12), which takes the conditional expectation *f* to be linear as in (13), and (ii) unpenalized fitting of model (8). We considered two settings, one in which model (12) holds and one in which it does not. Specifically, in both settings the latent response was of the form

(20) $$\ell_{\mathrm{ij}}=0.17-0.15\;\mathrm{sign}\left({\tilde{x}}_{i}-\tilde{x}\right){\left|{\tilde{x}}_{i}-\tilde{x}\right|}^{p}-0.72\;\mathrm{sign}\;\left(x_{\mathrm{ij}}-{\tilde{x}}_{i}\right){\left|x_{\mathrm{ij}}-{\tilde{x}}_{i})\right|}^{p}+u_{i}+\varepsilon_{\mathrm{ij}}.$$

For setting 1 we took p=1, yielding $\ell_{\mathrm{ij}}=0.17-0.15\left({\tilde{x}}_{i}-\tilde{x}\right)-0.72\left(x_{\mathrm{ij}}-{\tilde{x}}_{i}\right)+u_{i}+\varepsilon_{\mathrm{ij}}$, a special case of model (12). For setting 2 we took p=1/3.

The simulated data were designed to be broadly similar to the experience sampling data of Baumeister et al. (2020), and the coefficients in (20) were chosen to be representative of our results with that data set. In each of 100 replicates the data consisted of 6000 pairs $\left(x_{\mathrm{ij}},y_{\mathrm{ij}}\right)\in {\left\{1,2,\ldots ,7\right\}}^{2}$ representing observation $j\in \left\{1,\ldots ,15\right\}$ from individual $i\in \left\{1,\ldots ,400\right\}$. The individual‐*i* median predictor value ${\tilde{x}}_{i}$ was 1, 2, 3, 4, 5, 6, 7 with frequencies 16, 46, 85, 106, 85, 46, 16, respectively, and the overall median was $\tilde{x}=4$. Given the predictor values, the response *y*_*ij*_ was generated by first drawing a latent response $\ell_{\mathrm{ij}}$ from model (20) with i.i.d. standard normal $u_{i}$ and i.i.d. standard logistic $\varepsilon_{\mathrm{ij}}$, and then determining $y_{\mathrm{ij}}$ by (2) with $\left(\theta_{1},\ldots ,\theta_{6}\right)=\left(-1,1,3,5,7,9\right)$.

### Results

**FIGURE 2:**
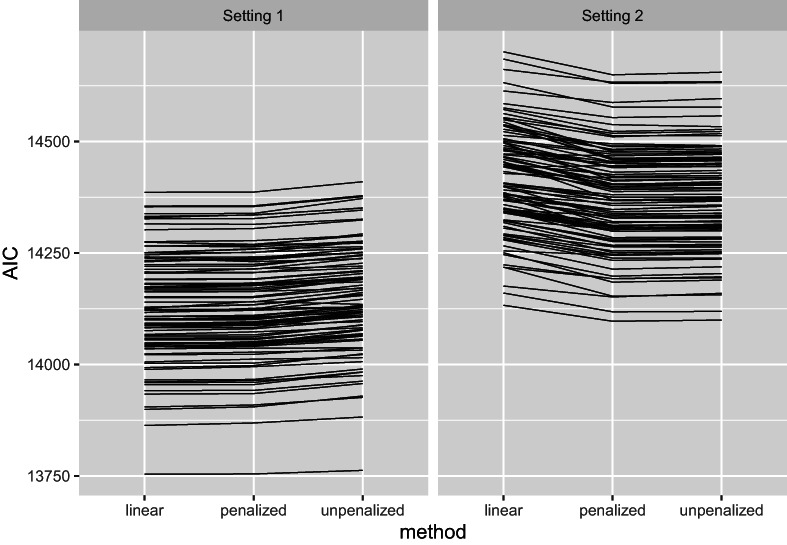
AIC attained by the three methods with data simulated from (20) with p=1 (setting 1, true f linear) and p=1/3 (setting 2, f nonlinear).

Figure 2 displays the values of the Akaike information criterion (AIC) attained by the three methods: linear (model (12)), penalized, and unpenalized. In setting 1, since the linear model (12) holds, this model attains the lowest AIC in 94 of the 100 replicates. But for the penalized fit, the data‐driven tuning method described in Section Optimal selection of the shrinkage parameter tends to result in a large λ, resulting in a near‐linear fit (see Section Effective degrees of freedom) and AIC values that are only slightly higher than those for the linear model. The unpenalized model does markedly less well, with a higher AIC than the penalized model in 99 of 100 replicates. For setting 2, by contrast, the linear model is clearly outperformed and attains the highest AIC in 99 of the 100 replicates, while the penalized model attains the lowest AIC in 84 replicates. In summary, the penalized fit attains the best overall performance: it is best when the true *f* is nonlinear, and near‐optimal even when *f* is linear.

**FIGURE 3:**
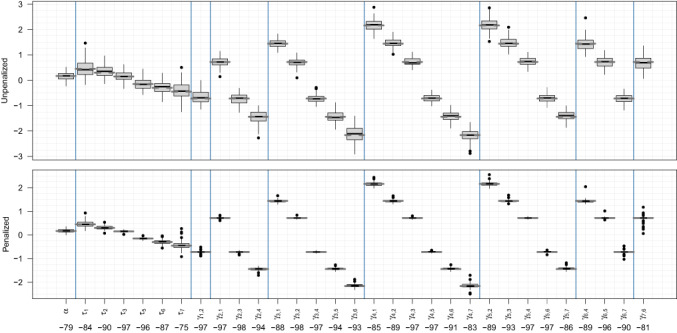
Box plots of coefficient estimates from simulation setting 1 without penalization (above) and with penalization (below), with true values given by dashed grey lines. Shown along the bottom are the percentage differences in mean squared error for the penalized estimates relative to the unpenalized: 79% lower for α, 84% lower for $\tau_{1}$, and so on.

**FIGURE 4:**
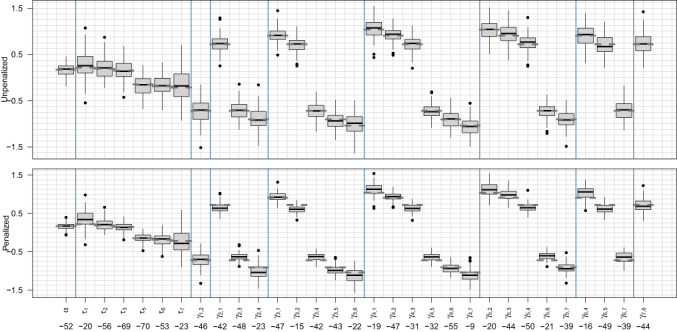
Coefficient estimate box plots as in Figure 3 but for setting 2.

Figures 3 and 4 compare the penalized and unpenalized fixed‐effect estimates via box plots, with dashed grey lines indicating the true coefficient values. In Figure 3 (setting 1), the generally narrower box plots in the lower plot imply that, in line with the AIC results in Figure 2, penalization noticeably improves the precision of the estimates. The disparity between the upper and lower plots is much less apparent in Figure 4 (setting 2). This is again consistent with the AIC results in Figure 2, and is to be expected, since penalization towards a linear model should be less effective when the true f is nonlinear. The bottom of each figure shows, for each parameter, the percentage difference in mean squared error for the penalized fits, relative to the unpenalized. These values confirm our visual impressions from the box plots, that penalization decreases mean squared error less dramatically for setting 2 than for setting 1.

### Effective degrees of freedom

For models fitted by maximizing a penalized likelihood such as (14), the notion of effective degrees of freedom (EDF; Wahba, 1983; Reiss et al., 2017) offers a means to quantify the shrinkage due to penalization. To gain further insight into the simulation results we consider the EDF associated with the fixed effects, where the EDF is partitioned among the model terms according to the approach of Wood (2017). For λ=0, the EDF equals the number of distinct fixed effects $\alpha ,\tau_{r},\gamma_{r,s}$ occurring in (5), or equivalently ∣K∣ for K defined in (10). As λ→∞, the EDF shrinks to 3, the number of fixed effects in the linear model (12).

**FIGURE 5:**
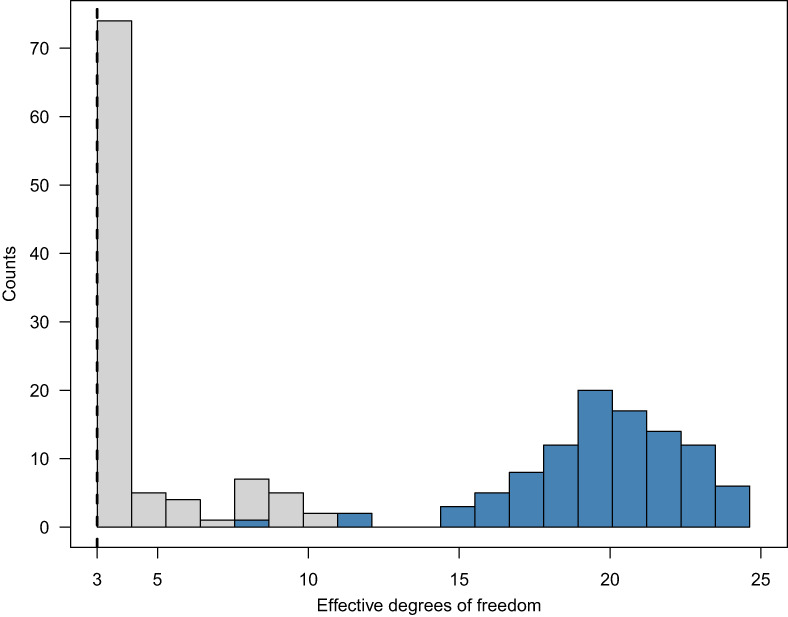
Effective degrees of freedom for the fixed‐effect terms of the penalized model fits in the simulation study, bounded below by 3 (the limit as λ→∞). Lighter histogram: Setting 1. Darker histogram: Setting 2.

In simulation setting 1, the true model is linear with respect to both trait and state, and thus has three fixed effects, whereas in setting 2 the true model is nonlinear. Figure 5 shows that the penalized model fits succeeded in detecting this distinction, in the sense that the fixed‐effect EDF was generally close to 3 in setting 1 but much higher in setting 2.

In summary, the penalized model fits’ EDF values, which are determined by λ as selected by the procedure of Wood et al. (2016) implemented in the *mgcv* package (see Section Optimal selection of the shrinkage parameter above), indicate that this procedure does a good job of letting the data decide how much shrinkage to apply (cf. Gertheiss et al., 2021).

## FITTING AND VISUALIZING MULTIPLE MODELS

### Parameter estimates

**FIGURE 6:**
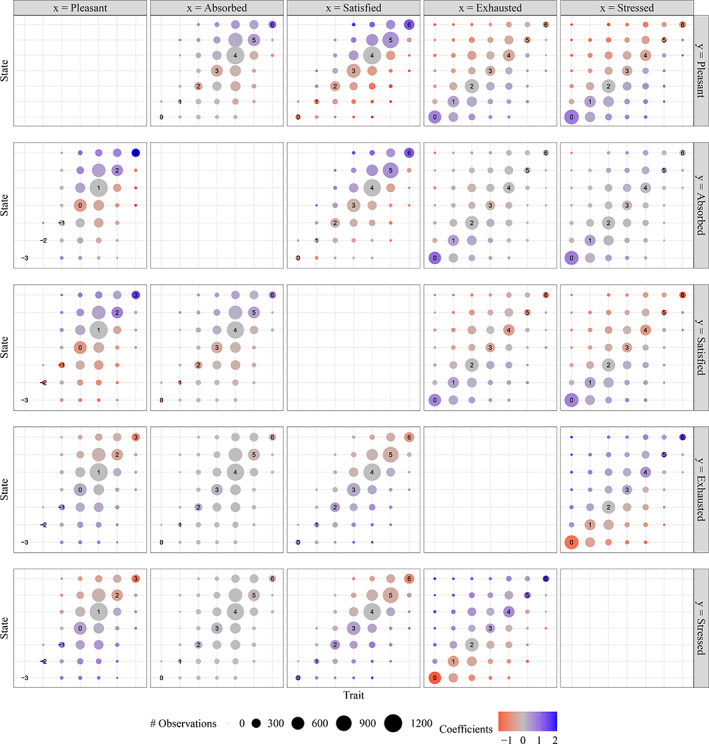
Standardized coefficient estimates for 20 penalized state‐trait models, represented as in the lower half of Figure 1.

Figure 6 shows coefficient estimates, with penalization, for regression of each of the five variables described in Section Motivating data set on each of the other four. The results are displayed as a grid or matrix of plots similar to those in the lower half of Figure 1. Each variable plays the role of response in one row of the grid, and the role of predictor in one column. Such a grid can be helpful for comparing the 20 models, but the raw coefficient estimates ${\hat{\tau }}_{r}$ and ${\hat{\gamma }}_{r,s}$ are not directly comparable across models since each model has different values of the cutpoints $\theta_{2},\ldots ,\theta_{6}$. The coefficients displayed in Figure 6 have therefore been standardized via division by the square root of expression (21) below, an estimate of the standard deviation of the latent response.

A positive relationship at the trait level implies that $\;\mathrm{sign}\;\left(\tau_{r}\right)=\mathrm{sign}\;\left(r-\tilde{x}\right)$ for each *r*, while a positive relationship at the state level implies $\;\mathrm{sign}\;\left(\gamma_{r,s}\right)=\mathrm{sign}\;\left(s-r\right)$ for each *r,s*. Similarly, a negative trait or state relationship implies $\;\mathrm{sign}\;\left(\tau_{r}\right)=\mathrm{sign}\;\left(\tilde{x}-r\right)$ or $\;\mathrm{sign}\;\left(\gamma_{r,s}\right)=\mathrm{sign}\;\left(r-s\right)$, respectively. As can be seen in Figure 6, the five variables can be divided into two groups, {pleasant, absorbed, satisfied} and {exhausted, stressed}, such that positive trait and state relationships are observed for pairs of variables in the same group (upper left‐ and lower right‐hand panels), while negative relationships are observed for between‐group variable pairs (upper right‐ and lower left‐hand panels). Consider, for example, the regression of exhausted on stressed, with coefficients displayed at lower right. The positive trait‐level association is seen in the increase along the main diagonal from ${\hat{\tau }}_{0}$ to ${\hat{\tau }}_{6}$. The positive ${\hat{\gamma }}_{r,s}$ values for s>r (upper left‐hand portion of the panel, meaning more stressed than usual), and the negative values for s<r, signify a positive state‐level association.

There are, on the other hand, a few examples in which the direction of association is less clear‐cut. For the regression of absorbed on pleasant, the association is overall positive at both the trait and state levels, but the positive values of several ${\hat{\gamma }}_{r,s}$ with s<r deviate from this pattern. In particular, ${\hat{\gamma }}_{0,s}>0$ for either s<0 or s>0, suggesting a possible association between being absorbed and having thoughts that are either more or less pleasant than usual (cf. the regression of absorbed on stressed). This result is admittedly quite a weak one, for which we can make no claim of statistical significance. Nevertheless, we mention it in order to highlight two points:

1. Such a non‐monotonic state‐level relationship between absorbed and pleasant is precluded by the more conventional model (12), but is allowed for by model (5).
2. The regression in the opposite direction, of pleasant on absorbed, indicates a more straightforward positive relationship at both the trait and state levels. Thus, although as a rule reversing the roles of *x* and *y* yields a qualitatively very similar fitted model (5), this example shows that the rule has exceptions.

### Latent *R* ^2^ for trait‐only and state‐trait models

As a measure of predictive power of the above models, we propose a form of *R* ^2^ on the latent scale, namely the ratio of $\hat{\;\mathrm{Var}\;}\left[\hat{f}\left({\tilde{x}}_{i},x_{\mathrm{ij}}\right)\right]$, the sample variance of the estimated fixed‐effects part of the model, to the estimated variance of $\ell_{\mathrm{ij}}$. While $\ell_{\mathrm{ij}}=f\left({\tilde{x}}_{i},x_{\mathrm{ij}}\right)+u_{i}+\varepsilon_{\mathrm{ij}}$ is latent rather than observed, the fact that the $\varepsilon_{\mathrm{ij}}$ are independent with unit variance suggests that we may take

(21) $$\hat{\;\mathrm{Var}\;}\left[\hat{f}\left({\tilde{x}}_{i},x_{\mathrm{ij}}\right)+{\hat{u}}_{i}\right]+1,$$

as our estimate of the variance of $\ell_{\mathrm{ij}}$ (i.e., the denominator in the ratio defining *R* ^2^). Similar versions of *R* ^2^ have been proposed by McKelvey and Zavoina (1975) for ordinal probit models and by Nakagawa and Schielzeth (2013) for generalized linear mixed models.

**FIGURE 7:**
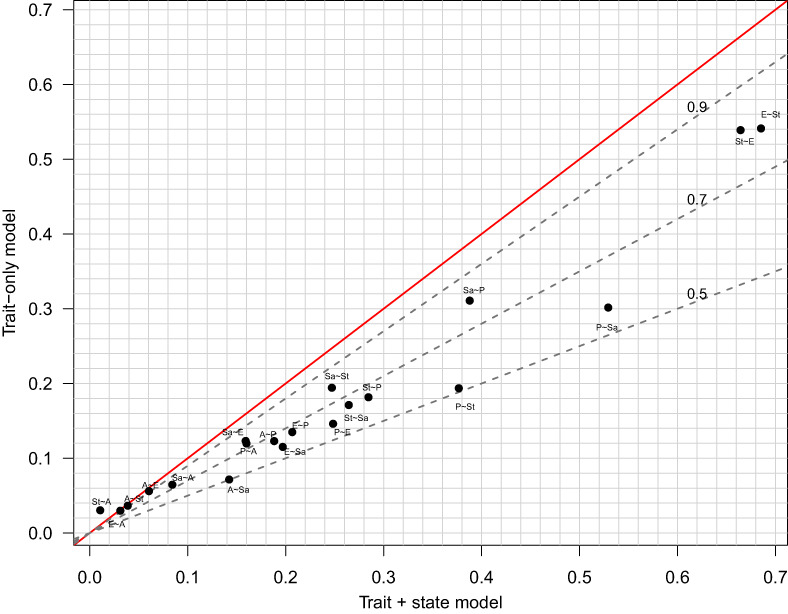
*R* ^2^ for trait‐only model (22) against the corresponding state‐trait model (5).

In Figure 7 we plot *R* ^2^ for each of the 20 instances of the ‘trait + state’ model (5) displayed in Figure 6, against *R* ^2^ for the corresponding ‘trait‐only’ model

(22) $$\ell_{\mathrm{ij}}=\alpha +\tau_{{\tilde{x}}_{i}}+u_{i}+\varepsilon_{\mathrm{ij}},$$

that is, model (5) with $\gamma_{{\tilde{x}}_{i},x_{\mathrm{ij}}}$ deleted. The point label ‘Sa~St’ denotes regression of satisfied on stressed, and so on for the other points. If, for a given response–predictor pair, we denote the *R* ^2^ values for the two models by $R_{\mathrm{TS}}^{2}$ and $R_{T}^{2}$, respectively, then the ratio $R_{T}^{2}/R_{\mathrm{TS}}^{2}$ can be used as an informal index of the relative importance of the predictor variable’s current (state) versus median (trait) value in the regression relationship. A high value of the ratio indicates the relationship is ‘trait‐driven’, whereas a low ratio suggests it is more ‘state‐driven’. Shown in the plot are dashed lines through the origin with slopes .5, .7 and .9; thus, for instance, those points lying between the lower two lines have $.5<R_{T}^{2}/R_{\mathrm{TS}}^{2}<.7$, indicating relatively state‐driven relationships. Based on this *R* ^2^ ratio, the relationships with pleasant as response (P~…) tend to be state‐driven, whereas those with satisfied as response (Sa~…) are relatively trait‐driven. This makes intuitive sense since life satisfaction is more stable than pleasantness of one’s current thoughts. Another pattern that emerges from Figure 7 is that models involving absorption (A), as either predictor or response, have consistently low *R* ^2^ values. In the lower left‐hand corner, the regression of stressed on absorbed is seen to have a higher *R* ^2^ for the trait‐only model; this is anomalous but can sometimes occur for penalized models, especially in cases such as this of a very weak association.

## SERIAL DEPENDENCE

A limitation of model (5) is that different responses for a given individual are treated as independent, conditional on the random subject effect; serial dependence is not modelled. To remedy this limitation we consider the model

(23) $$\ell_{\mathrm{ij}}=\alpha +\beta y_{i,j-1}+\tau_{{\tilde{x}}_{i}}+\gamma_{{\tilde{x}}_{i},x_{\mathrm{ij}}}+u_{i}+\varepsilon_{\mathrm{ij}},$$

that is, we augment model (5) by adding the previous value of the response *y* as a predictor. While this has the advantage of avoiding a questionable assumption of (conditionally) independent responses, it also has several disadvantages:

1. The first observation for each subject, or each day for each subject, must be omitted since there is no previous *y*.
2. This model treats lagged *y* as continuous, which is at odds with our general approach of treating both response and predictors as ordinal.
3. A model assuming a fixed effect *β* of lagged response is less appropriate when, as in our data set, the time points are irregularly spaced.

To assess whether the lagged‐response term is worth adding to the model despite these disadvantages, we refitted the 20 regressions of Section Fitting and visualizing multiple models with model (23). The results cannot be directly compared to the model (5) fits by AIC since, as noted above, some observations are dropped when fitting (23) and thus the sample sizes differ. However, since AIC is an estimate of the expected log‐likelihood for a new data set with the same predictor values (Davison, 2003), we can perform an approximate model comparison by dividing AIC by sample size for the two models and comparing the results. Including the lagged response as a predictor has negligible effect on this scaled AIC for the models with pleasant, absorbed, and satisfied as response, but reduces (i.e., improves) it somewhat for those with exhausted and stressed as response. The resulting estimates of state and trait effects appear virtually unchanged from those displayed in Figure 6.

## DISCUSSION

In this paper we have developed a state‐trait cumulative logistic model for settings in which both the response and the predictor are ordinal. Rather than requiring the latent response to depend linearly on (i) the person‐specific median and (ii) the deviation therefrom, as in (12), our proposed model (5) allows for nonlinear relationships. To avoid overfitting we impose a novel penalty that shrinks the fit towards linearity.

The methods of this paper are implemented in an R package called *store* (*s*tate‐*t*rait *o*rdinal *re*gression), which is publicly available at https://github.com/oseipep/store. The package allows person‐level covariates, such as demographic variables or personality scales, to be added to model (5). In some studies a person‐level variable, say a measure of well‐being, might be closely related to an intensively sampled variable, such as current positive mood. In such a case, the former and the median of the latter might be capturing roughly the same trait. Our covariate‐adjusted model could help to assess the relative contributions of these alternative trait measures, as well as that of the corresponding state measure.

Extending the model to allow multiple repeatedly measured ordinal predictors, with trait and state terms for each, is in principle straightforward. This would, however, call for a separate penalty for each of these predictors, and thus markedly increase the computational burden. We may include such a capability in future versions of the package. For now, we note that a collection of single‐predictor models, such as the 20 models presented in Section Fitting and visualizing multiple models for the data of Baumeister et al. (2020), can yield quite interesting insights.

Penalty functionals other than (16) may be useful in particular applications. For example, a penalty that smooths across neighbouring cells $\left(r,s\right)$ might be preferable to ad hoc merging of small cells. Here again, though, the benefit must be balanced against the computational burden implied by multiple penalties.

While model (5) concerns only the relationship between current *x* and current *y*, one is sometimes interested in lagged associations, which may offer stronger evidence of a causal connection between *x* and *y*. As noted in Section Serial dependence in connection with adding lagged *y* to model (5), simply adding one or more previous values of *x* to the model would be problematic for typical ESM data sets since the time lag between observations is non‐uniform. We are currently developing a novel solution to this problem that models the effect of previous *x* as a smooth function of the time lag.

In some applications it might be of interest to add random slopes to model (5), although our current implementation allows only random intercepts. Other possible extensions of the model include incorporating temporal development or change (Curran & Bauer, 2011; Wang & Maxwell, 2015) and multiple responses as in the integrated trait‐state model of Hamaker et al. (2007).

This paper has been concerned with asymmetric (regression) relationships between two ordinal variables. Alternatively, one might wish to examine symmetric (covariance/correlation) relationships among two or more ordinal variables. For partitioning such relationships into between‐ and within‐person components, a Bayesian approach based on Markov chain Monte Carlo (MCMC) may be required. The MCMC implementation of Hadfield (2010) is one possible route in that direction.

## AUTHOR CONTRIBUTIONS

**Prince P. Osei:** Data curation; formal analysis; investigation; methodology; software; validation; visualization; writing – original draft; writing – review and editing. **Philip Reiss:** Conceptualization; data curation; formal analysis; funding acquisition; investigation; methodology; project administration; software; supervision; validation; writing – original draft; writing – review and editing.

## CONFLICT OF INTEREST

All authors declare no conflict of interest.

### OPEN RESEARCH BADGES

This article has earned Open Data and Open Materials badges. Data and materials are available at https://github.com/oseipep/store.

## Data Availability

The data that support the findings of this study are openly available on the Open Source Framework at https://osf.io/9uytp/.

## INFREQUENT TRAIT‐STATE PAIRS

For some pairs $\left(r,s\right)$, there may be very little data with which to estimate $\gamma_{r,s}$, that is, very few observations with x=s for participants with median *x* equal to *r*. This results in estimates ${\hat{\gamma }}_{r,s}$ that are unstable and that, in some cases, may diverge to ±∞. This problem can be avoided by appropriately merging pairs. For our analyses of the data of Baumeister et al. (2020) reported in Sections Pleasant thoughts and stress revisited and Fitting and visualizing multiple models, if the pair $\left(r,s\right)$ had fewer than 10 observations, it was collapsed into $\left(r,s+1\right)$ for s<r and $\left(r,s-1\right)$ for s>r. This was repeated as needed until all pairs had at least 10 observations.

## The matrix **P**

Here we derive a matrix **P** such that $\;\zeta^{T}P\;\zeta =J^{*}\left(f\right)$, the penalty functional given in (16). As noted in Section Motivation, the vector ζ consists of the coefficients α, $\tau_{r}$ for each r, and $\gamma_{r,s}$ for each $\left(r,s\right)$ occurring in the data with r≠s. First we rewrite (17)–(19) in terms of the regression coefficients as

(24)

By (24), for each $\left(r,s\right)$ such that $\Delta_{\mathrm{rr}}f\left(r,s\right)$ is defined, there is a vector $v_{r,s}$ such that $v_{r,s}^{T}\;\zeta =\Delta_{\mathrm{rr}}f\left(r,s\right)$. Let $Q_{\mathrm{rr}}$ be the matrix obtained by stacking all the row vectors $v_{r,s}^{T}$. Then $\;\zeta^{T}Q_{\mathrm{rr}}^{T}Q_{\mathrm{rr}}\;\zeta =\sum {\left[\Delta_{\mathrm{rr}}f\left(r,s\right)\right]}^{2}$. We can similarly define $Q_{\mathrm{rs}},Q_{\mathrm{ss}}$ such that $\;\zeta^{T}Q_{\mathrm{rs}}^{T}Q_{\mathrm{rs}}\;\zeta =\sum {\left[\Delta_{\mathrm{rs}}f\left(r,s\right)\right]}^{2}$ and $\;\zeta^{T}Q_{\mathrm{ss}}^{T}Q_{\mathrm{ss}}\;\zeta =\sum {\left[\Delta_{\mathrm{ss}}f\left(r,s\right)\right]}^{2}$. Thus $P=Q_{\mathrm{rr}}^{T}Q_{\mathrm{rr}}+2Q_{\mathrm{rs}}^{T}Q_{\mathrm{rs}}+Q_{\mathrm{ss}}^{T}Q_{\mathrm{ss}}$ implies $\;\zeta^{T}P\;\zeta =J^{*}\left(f\right)$, as required.
